# 55256 ReacStick: From Conception to Commercialization

**DOI:** 10.1017/cts.2021.510

**Published:** 2021-03-30

**Authors:** Alec Bernard, Ian Richarson, James Richardson

**Affiliations:** 1 University of Michigan Medical School; 2 Benjamin Dalusma, MIT Sloan School of Management; 3 Professor of Physical Medicine and Rehabilitation, University of Michigan

## Abstract

ABSTRACT IMPACT: ReacStick concussion testing and monitoring can serve as a 'vital sign for the brain', allowing for an immediate, objective assessment on the field or at the bedside. This project examines the entrepreneuship process from invention to commercialization. OBJECTIVES/GOALS: ReacStick is the first objective, portable, measure of concussion likelihood and severity and uses simple and complex reaction time testing. We detail the entrepreneurship process from product invention through its current mid-stage (patented, 20+ publications, etc.) to future commercialization for diverse applications. METHODS/STUDY POPULATION: ReacStick was invented in 2010 and underwent extensive testing and validation of the underlying innovations. The regulatory landscape of the product was examined, and 510(k) was found to be the best pathway. Competitive analysis was done examining alternative products and comparing against the current gold standards. A customer discovery process was undertaken, and stakeholders were interviewed for feedback and iteration. Testing and validation were completed with athletes, older adults, and people taking medications. An overview of the necessary commercialization concepts is: market opportunity/monetization, intellectual property considerations, regulatory processes, commercialization plan. RESULTS/ANTICIPATED RESULTS: ReacStick accurately predicts concussion and time to recovery and was patented through UM Tech Transfer in 2010, with 10 years currently remaining on the patent. Through customer discovery processes, athletics was determined to be the most viable first market to enter. Next steps include seeking additional patent protection, capital investors, delivery of minimum viable product followed by iteration and improvement for military, emergency medicine and acute care use. The current remaining timeline involves 12-18 months to commercialization and includes regulatory approval, additional patent protection, collaboration with regulatory consultants, capital fundraising and product production. DISCUSSION/SIGNIFICANCE OF FINDINGS: The research team has gone through a lengthy process toward commercialization of ReacStick. Proof of concept and extensive validation of the underlying technology have been completed and the regulatory process has been mapped. Our experience can serve as a model of many of the steps and challenges that lie on the path from lab to sale to end users.

